# T-rex: standardized analysis of germline variants in whole-exome sequencing trios

**DOI:** 10.1038/s41598-026-67762-w

**Published:** 2026-08-31

**Authors:** Sara-Luisa Reh, Carolin Walter, Judith Lohse, Tabita Ghete, Markus Metzler, Julia Hauer, Franziska Auer

**Affiliations:** 1 School of Medicine and Health, Department of Pediatrics, Technical University of Munich, Munich, Germany; 2 Bavarian Cancer Research Center (BZKF), München, Germany; 3 Institute of Medical Informatics, University of Muenster, 48149 Muenster, Germany; 4 Pediatric Hematology and Oncology, Department of Pediatrics, Faculty of Medicine Carl Gustav Carus, University Hospital, Dresden University of Technology (TUD), Dresden, Germany; 5 Department of Pediatrics and Adolescent Medicine, University Hospital Erlangen, Erlangen, Germany; 6 Pediatric Oncology Network Bavaria, KIONET, Bavaria, Germany; 7 Bavarian Cancer Research Center (BZKF), Erlangen, Germany; 8 German Center for Child and Adolescent Health (DZKJ), partner site, Munich, Germany

**Keywords:** Genetic predisposition, Trio-based sequencing, Germline variants, Rare disease, WES analysis platform, Cancer, Computational biology and bioinformatics, Genetics, Medical research

## Abstract

Whole-exome sequencing (WES) enables the identification of rare germline variants contributing to pediatric diseases. Trio-based sequencing, comparing affected children with their parents, is particularly effective for rare disease genetics. However, WES data analysis requires bioinformatics expertise, varies across institutions, and is often incompatible with clinical workflows. We developed T-Rex (**T**rio **R**are variant analysis of **EX**omes), a cross-platform desktop application that enables the standardized and local analysis of WES germline Trio data without the need for programming knowledge. T-Rex integrates state-of-the-art tools for alignment, dual-variant calling (GATK HaplotypeCaller + VarScan2), annotation (SNPEff/SNPSift), rare-variant filtering based on population frequencies (gnomAD), and family-based statistical testing, including the Transmission Disequilibrium Test with multiple-testing correction. Benchmarking of the dual-caller strategy on the Genome in a Bottle Ashkenazim Trio demonstrates high precision (99.2%) while maintaining robust sensitivity (91.1%). User testing (*n* = 13) confirmed quick learning across clinicians and researchers. Application to a cohort of *n* = 121 pediatric cancer Trio datasets, filtering for rare protein-coding variants (MAF ≤ 0.1% in gnomAD v4.1), validated all assessable previously reported pathogenic variants. Overall, T-Rex enables clinicians to robustly analyze WES Trio data in compliance with data protection regulations without requiring additional software licenses. As one of the first platforms for comprehensive WES Trio analysis that requires no programming expertise while providing reproducible, end-to-end workflows for clinical genomics, T-Rex facilitates collaborative research between clinics and reduces reliance on external providers.

**Supplementary Information:**

The online version contains supplementary material available at https://doi.org/10.1038/s41598-026-67762-w.

## Introduction

Rare diseases collectively affect millions of individuals, yet many rare conditions remain without an identified molecular basis despite advances in genomic medicine^1,2,3^. Progress in rare disease genomics is constrained by the difficulty of recruiting sufficiently large patient cohorts, which in turn limits the generation of statistically robust results and the application of artificial intelligence approaches. As individual centers typically encounter few affected individuals, meaningful studies rely on collaboration across institutions. Such collaboration is also essential for the delivery of high-quality genomic services and improved patient care^4,5^. However, germline sequencing data constitute sensitive personal data, as they include heritable information with implications for both individual privacy and familial relationships. Thus, legal and ethical constraints on data protection often prevent the sharing of raw patient-level sequencing data, making decentralized, local analysis essential^6^.

Since approximately 75% of rare diseases manifest in childhood, Trio studies, which sequence affected children along with both parents, have become a cornerstone for identifying pathogenic variants^7,8^. By directly comparing transmitted and non-transmitted alleles within families, trio-based sequencing both reduces false positives and controls for population stratification, making it well-suited for small, heterogeneous cohorts assembled through multi-center collaborations^9,10,11^. This is particularly relevant for childhood cancer, which arises largely in the absence of lifestyle-related exposures, and for which germline genetic predisposition has already been implicated in up to 15% of cases^12,13^.

Despite the increasing availability of whole-exome sequencing (WES), clinical scientists often lack access to standardized, easy-to-use, and openly available tools for Trio WES analysis^14,15^. Existing open-source WES analysis pipelines commonly depend on command-line interfaces, operating-system-specific dependencies, or container platforms such as Docker or Nextflow, which require substantial programming knowledge, limiting adoption in laboratories without dedicated bioinformatics support^16,17,18,19,20^. While commercial trio-analysis platforms are available^21,22^, licensing costs and proprietary implementations may limit accessibility in some academic and clinical settings^23^.

To address these challenges, we developed T-Rex (**T**rio **R**are variant analysis of **EX**omes), a standalone, cross-platform desktop application providing a standardized WES pipeline for Trio studies, including rare-variant filtering and statistical testing. T-Rex enables clinicians to securely perform local WES Trio analyses without programming knowledge or sharing raw sequencing data. T-Rex supports batch execution and job resumption and, for advanced users, can additionally be operated in headless mode and parallelized on server environments when required. We demonstrate its utility using a Trio cohort of *n* = 121 children with cancer and their parents.

## Methods

### Software architecture

T-Rex is implemented in Python with a Tkinter/CustomTkinter graphical user interface^24,25^ and follows a Model–View–Controller architecture. The backend executes integrated Bash scripts for alignment, variant calling, and annotation, ensuring reproducible analyses across macOS, Linux, and Windows using only free and open-source software (FLOSS). T-Rex is distributed as a standalone desktop application, enabling straightforward installation without complex dependencies, administrative privileges, or command-line interaction. The platform was designed with a strong focus on usability, informed by regular feedback from clinicians and scientists. The interface is deliberately kept simple, with analysis options minimized, and interactive elements restricted during analysis to prevent accidental termination. Extensive in-platform instructions further support users without bioinformatics experience.

### WES processing pipeline

**Fig. 1:**
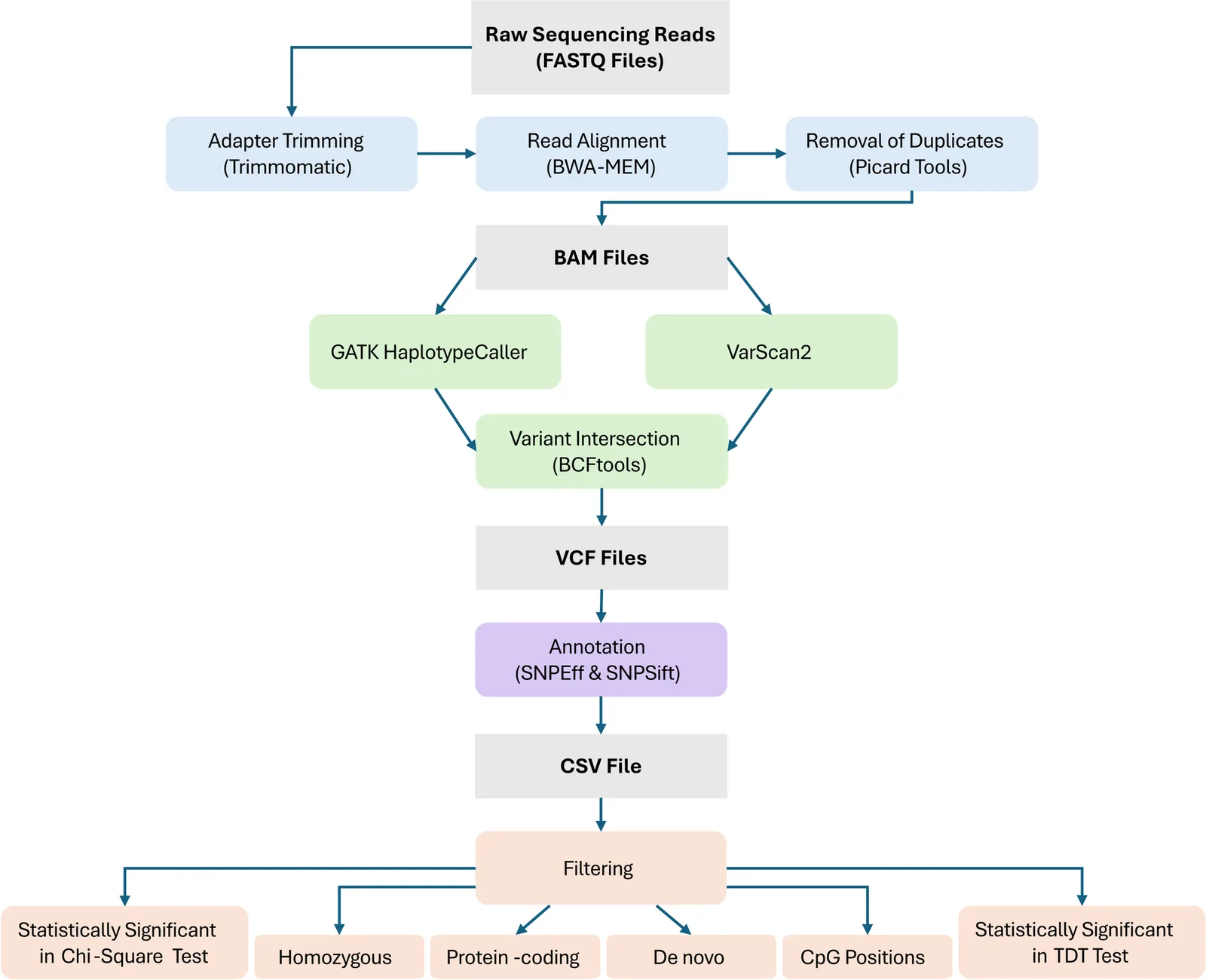
Flowchart illustrating the implementation of the WES data analysis pipeline.

The WES analysis pipeline processes short-read, paired-end Illumina data from Trio studies to identify germline variants in accordance with current software recommendations and best-practice guidelines, with optional support for single-end data. Users can initiate the pipeline at the alignment, variant calling, annotation, or filtering stage, enabling the analysis of FASTQ, BAM, VCF, CSV, and TSV input formats. A flowchart summarizing the pipeline implementation and the specific software tools used is shown in Fig. 1.

#### Pre-processing and alignment

Following the current best practices for short-read alignment^14,26,27^, Illumina paired-end reads undergo:

- Adapter trimming with Trimmomatic^28^,
- Alignment to GRCh38 primary assembly using BWA-MEM^29^,
- Duplicate removal with Picard^30^,
- BAM indexing with SAMtools^17^.

#### Dual variant calling

Following evidence that combining complementary variant callers improves accuracy^14,27,31,32^, T-Rex integrates two algorithms with distinct methodological strengths for germline variant detection. Since GATK HaplotypeCaller^16^ and VarScan2^33^ exhibit complementary performance characteristics^32,34^, have both been validated for use with trio-based germline data^14,35^, and have been recommended for ensemble use by prior studies^14,34^, we implemented a dual-caller strategy integrating both tools for variant detection. Variant call sets were intersected using BCFtools^17^, retaining only variants independently identified by both callers to increase precision.

#### Annotation and filtering

Annotation is performed using SNPEff^36^ to predict functional impact, and SNPSift^37^ to retrieve allele frequencies from the Genome Aggregation Database (gnomAD) v4.1, including the overall gnomAD frequency, the maximum population allele frequency (PopMax), ancestry-specific allele frequencies for the European and non-Finnish European populations^38^, as well as to assess variant pathogenicity based on ClinVar^39^.

After annotation, users can apply additional filtering options, including maximum allele frequency (default ≤ 1%) for a selected reference population, protein-coding variants only, homozygous or *de novo* variants only, variants located at CpG positions, and variants significant in either the Chi-Square Test or the Transmission Disequilibrium Test (TDT). When multiple filters are applied, only variants meeting all specified criteria will be included in the results.

#### Statistical testing

T-Rex implements two classes of statistical tests: (1) case–population comparison, using Fisher’s exact test when expected counts are ≤ 5 and Pearson’s χ² test for larger counts^40,41,42^; and (2) case–parent comparison, using the TDT, which applies McNemar’s test or exact binomial testing based on transmitted versus non-transmitted alleles from heterozygous parents^10,43,44,45^. Multiple-testing correction is performed using the Bonferroni method, as recommended for genome-wide rare variant analysis^11,46,47^.

### Benchmarking with the GIAB Ashkenazim trio

The dual-variant calling strategy was benchmarked on WES data from the Ashkenazim Trio reference samples (HG002 [child], HG003 [father], HG004 [mother]) provided by the Genome in a Bottle (GIAB) Consortium^48^. Variant calling was performed using the integrated dual-caller workflow, combining GATK HaplotypeCaller v4 and VarScan2 in trio mode. Performance was assessed against the GIAB high-confidence truth set for HG002 (child), restricted to the exome target intervals. Precision, recall, and F1-score were calculated with RTG Tools v3.12^49^. Benchmarking results from the consensus approach were compared to those obtained using GATK HaplotypeCaller v4 and VarScan2 individually, restricted to the same genomic regions.

### Performance and user testing

T-Rex was evaluated following standard software testing guidelines^50,51^, including software, performance, and user acceptance testing.

Time and space complexity of individual pipeline components and the complete workflow were assessed on a system with 8 GB of RAM and 8 CPUs, using validated synthetic test sequences. In addition, wall-clock runtime and peak memory usage were measured on a system with 128 GB of RAM and 8 CPUs, using 121 real Trio WES samples.

User acceptance testing involved 13 participants (clinicians, research scientists, and doctoral candidates), aged 22–62 years (mean: 33.2 years), who completed structured tasks followed by semi-structured interviews, with task completion times recorded (**Supplemental Sect.  1**). Platform design was iteratively refined based on user feedback.

### Trio cohort analysis

Analyzed germline DNA was derived from fibroblasts or blood and was previously sequenced at > 100x coverage on Illumina NovaSeq^52^. The raw FASTQ files were processed by applying T-Rex’s analysis pipeline, filtering for protein-coding variants with a maximum population (PopMax) allele frequency of less than 0.1% according to gnomAD v4.1. Variant pathogenicity was assessed based on ClinVar annotations. The results were then compared with those reported by *Friedrich et al.* (2023) by evaluating whether the 14 variants classified as (likely) pathogenic by clinical geneticists in that study were also detected in the T-Rex analysis output.

### Patient consent

The cohort data used for re-analysis were derived from patients younger than 19 years of age who were recruited unselectively at the Pediatric Oncology Department of the University Hospital and Faculty of Medicine Carl Gustav Carus, Technische Universität Dresden, between 2019 and 2021. Male and female participants were included without selection bias for sex or gender. The study with all experimental protocols was reviewed and approved by the Ethics Committee at the Technische Universität Dresden, Dresden, Germany, under the study title “Keimbahnmutationen bei krebskranken Kindern und Jugendlichen” (reference number EK181042019; final assessment dated 03 September 2019). All methods were carried out in accordance with relevant guidelines and regulations and with the principles of the Declaration of Helsinki. Informed consent was obtained from all subjects and/or their legal guardian(s).

## Results

### Linear time and constant space complexity

**Fig. 2:**
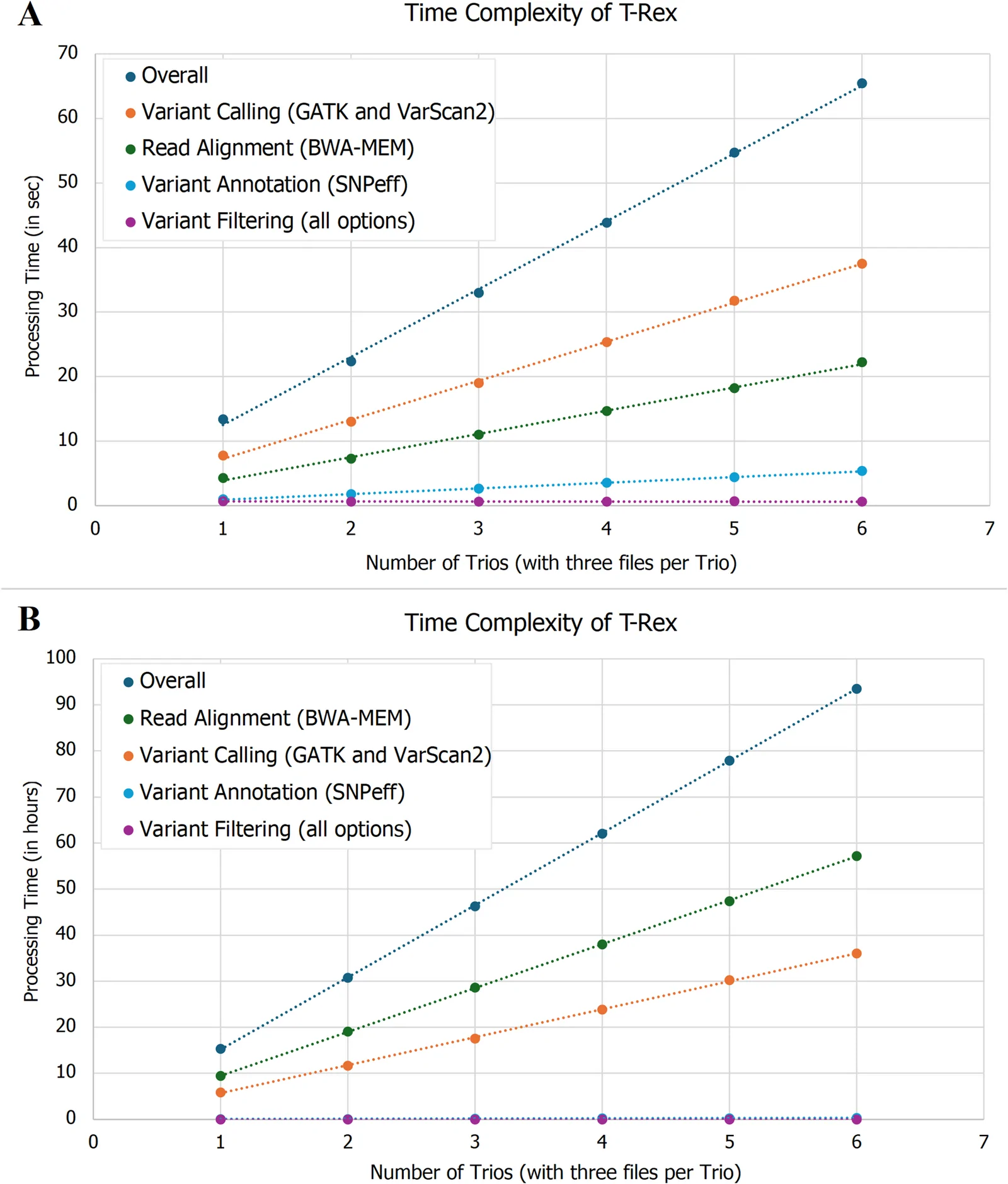
Time complexity of T-Rex on two different systems. A. Time complexity of validated synthetic test sequences on a system with 8 GB RAM and 8 CPUs. Variant calling with GATK HaplotypeCaller is slowed when RAM is below 16 GB, which explains why variant calling is slower than alignment on this system. B. Runtime of real WES Trio datasets measured on a server with 8 CPUs and a peak memory usage of 16 GB.

The time complexity across the pipeline was linear in cohort size (*O(n)*) (Fig. 2), while the space complexity remained constant (*O(1)*).

Memory usage adapted to system specifications but did not exceed 16 GB. When less than 16 GB of RAM was available, the runtime of GATK HaplotypeCaller increased, leading to longer overall analysis times, whereas the execution time of other pipeline components remained unaffected. On a server equipped with 8 CPUs and peak memory usage of 16 GB, processing 121 real-world Trio WES datasets required 15.3±0.7 h on average.

### Platform operation is easily learned

**Fig. 3:**
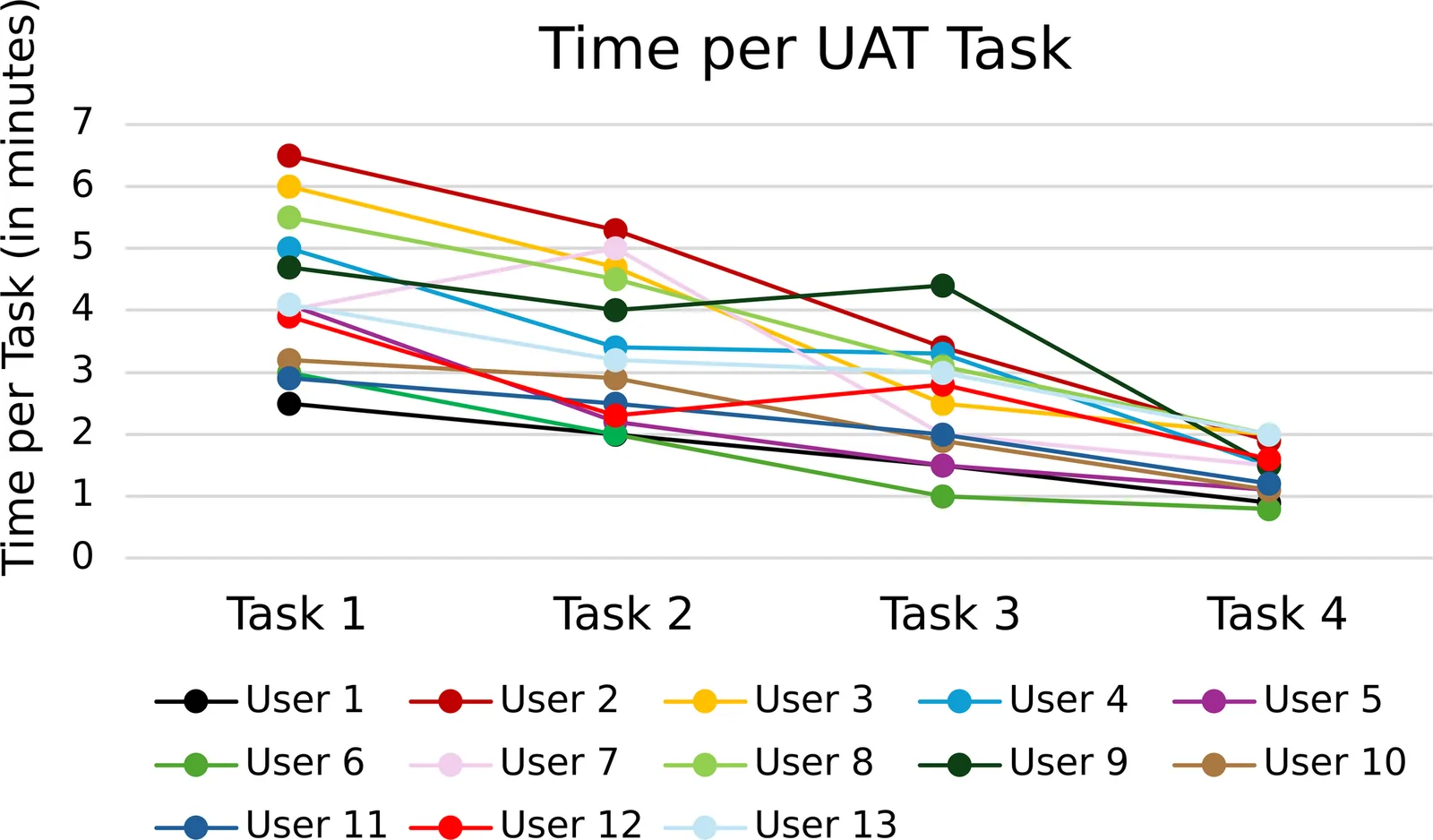
Comparison of task completion times during UAT across different users. Although task difficulty increased progressively, the time required per task decreased, indicating a learning effect. UAT, user acceptance testing.

All 13 participants learned to operate the platform in under 10 min, regardless of age or technical background. User feedback and iterative design adaptations further improved usability (**Supplemental Table S3**), enabling non-bioinformaticians to correctly and independently initiate Trio WES data analysis in under 2 min by the end of the study (Fig. 3).

### High precision of dual-caller workflow on GIAB

To assess the accuracy of the dual-variant calling workflow, variant calls from WES data of the Ashkenazim Trio (HG002–HG004) were benchmarked against the GIAB high-confidence truth set for HG002 (child) within the WES target regions (Table 1).

The dual-caller consensus approach (GATK v4 + VarScan2), which retained only variants identified by both tools, achieved a high precision of 99.2% with only 175 false positives while maintaining a sensitivity of 91.1%, resulting in an F1-score of 95.0%. Individually, GATK HaplotypeCaller v4 exhibited higher sensitivity (95.5%) but lower precision (96.0%) due to 916 false positives, whereas VarScan2 showed slightly lower precision (99.0%) but similar sensitivity (91.1%) with 204 false positives. These results demonstrate that the dual-caller strategy prioritizes high-confidence variant calls, substantially reducing false positives compared to the individual callers, while maintaining acceptable sensitivity, making it advantageous for clinical and research applications where precision in rare variant detection is critical.

**Table 1:** Performance of dual-variant calling on GIAB Ashkenazim Trio WES data for HG002 (child). Evaluation was performed in GIAB high-confidence regions intersected with WES target regions. True positives (TP), false positives (FP), false negatives (FN), precision, sensitivity, and F1-score are reported for individual callers (GATK HaplotypeCaller v4, VarScan2 in trio mode) and the dual-caller consensus workflow (GATK v4 + VarScan2). The dual-caller approach reduces false positives while maintaining comparable sensitivity.

Variant Calling	TP	FP	FN	Precision	Sensitivity	F1-Score
GATK v4 + VarScan2	21,202	175	2079	99.2%	91.1%	95.0%
GATK v4	22,229	916	1042	96.0%	95.5%	95.8%
VarScan2	21,218	204	2063	99.0%	91.1%	94.9%

### Trio cohort characteristics

**Fig. 4:**
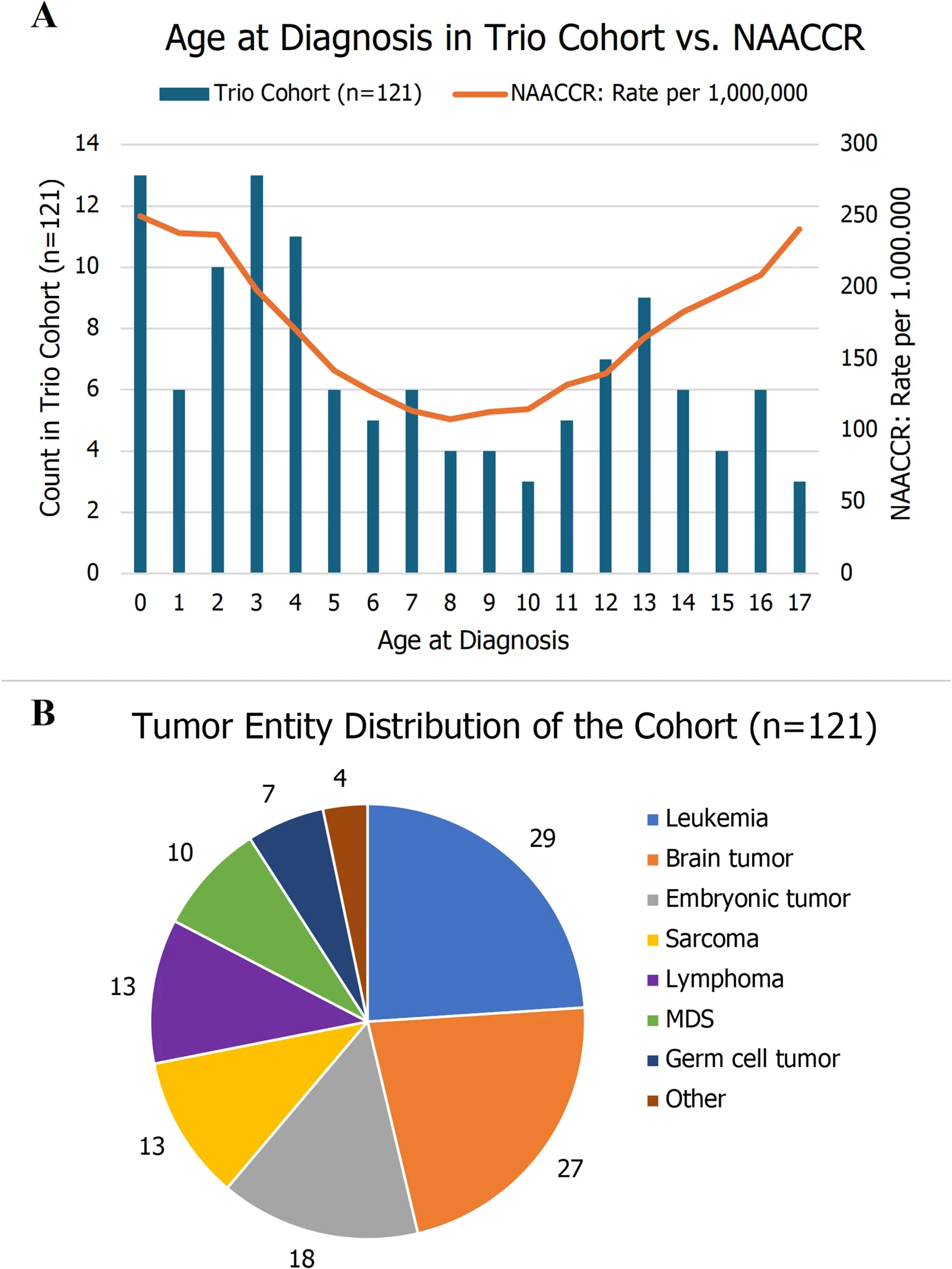
Characterisation of the Trio cohort. A. Comparison of the age at diagnosis of childhood cancer between the Trio cohort (*n* = 121) and the North American Association of Central Cancer Registries (NAACCR) (based on data from the National Cancer Institute (2024))^77^. The NAACCR documents the incidence of childhood cancer in 75% of all children in the USA (x)^77^. B. Tumor entity distribution of the Trio cohort (*n* = 121).

To assess the end-to-end applicability of T-Rex and its analytical concordance with previously reported findings in a real-world dataset, we re-analyzed raw WES data from *n* = 121 pediatric cancer Trios previously reported by our group as part of the *Friedrich et al. (2023)*^52^ cohort, which originally comprised *n* = 139 Duo and Trio datasets^52^. Although several underlying bioinformatics tools overlapped with those used in the original study-specific analysis, the previous analysis was not performed using the T-Rex application. The present re-analysis therefore evaluated whether the newly integrated and standardized T-Rex workflow could reproducibly recover the previously reported clinically relevant variants directly from the raw sequencing data. Because the original publication did not report the duo or trio status at the individual-case level, the exact composition of the trio-only subset, including its distributions according to age at diagnosis and tumor entity, cannot be reconstructed from the published data. We therefore provide the characteristics of the specific trio-only cohort analyzed in the present study in Fig. 4.

The age at cancer diagnosis in this trio-only cohort (*n* = 121) ranged from one month to 17.7 years, with a mean age of 7.1 years. The age at diagnosis is slightly bimodally distributed, with peaks in infancy (age 0 to 4) and adolescence (age 11 to 16) (Fig. 4A).

The most common cancer type in the cohort was leukemia (*n* = 29), with the majority being B-cell acute lymphoblastic leukemia (*n* = 22), followed by acute myeloid leukemia (*n* = 4) and T-cell acute lymphoid leukemia (*n* = 4) (Fig. 4B). Brain tumors were the second most common cancer type in the cohort (*n* = 27), followed by embryonic tumors (*n* = 18), sarcomas (*n* = 13), lymphomas (*n* = 13), myelodysplastic syndrome (MDS) (*n* = 10), and germ cell tumors (*n* = 7). The “other” tumor category includes two melanomas, one carcinoid, and one case of histiocytosis.

### T-rex detects previously reported actionable variants

First, we compared variant detection between T-Rex and *Friedrich et al. (2023)*^52^. Of the 13 (likely) pathogenic variants assessable within our dataset, T-Rex detected all 13 (Table 2). Two additional variants reported by *Friedrich et al. (2023)*^52^ were not expected to be detected: one originated from a Duo dataset (father-child) not included in our analysis, and one was an intronic variant located outside the WES target regions defined in the BED file.

**Table 2:** Comparison of variant detection between T-Rex and *Friedrich et al. (2023)*^52^. Of the 13 variants that were assessable within the dataset analyzed in this study, T-Rex successfully detected all 13. Two variants reported by *Friedrich et al. (2023)*^52^ were not expected to be detected: one originated from a Duo dataset not included in the analysis, and one was an intronic variant located outside the WES target regions defined in the BED file. These target regions can be expanded by the user if desired.

Cancer Type	Gene	Variant	Effect	Detected by T-Rex
MDS	*ERCC6L2*	NM_001010895.2:c.1963C>T	stop_gained	yes
	*MECOM*	NM_004991.4:c.2861G>C	missense	yes
	*WRAP53*	NM_001143990.1:c.1192C>T	missense	yes
	*WRAP53*	NM_001143990.1:c.904G>T	missense	yes
	*BRCA2*	NM_000059.3:c.6269del	frameshift	yes
Neuroblastoma	*BARD1*	NM_000465.4:c.951_957del	frameshift	yes
	*BARD1*	NM_000465.4:c.1932_1933del	frameshift	yes
Brain tumor	*BRCA2*	NM_000059.3:c.4214_4218del	frameshift	yes
	*ATM*	NM_000051.3:c.3085dup	frameshift	yes
	*TSC1*	NM_000368.4:c.1963C>T	stop_gained	yes
	*NF1*	*Intronic variant (not covered by the WES target BED file)*		
	*TP53*	NM_000546.5:c.499C>T	stop_gained	yes
Leukemia	*BRCA2*	NM_000059.3:c.3G>A	start_lost	yes
	*CHEK2*	NM_001005735.1:c.1229del	frameshift	yes
	*POT1*	*Duo only (not in analyzed Trio data set)*		

### Rare variant landscape in the trio cohort

**Fig. 5:**
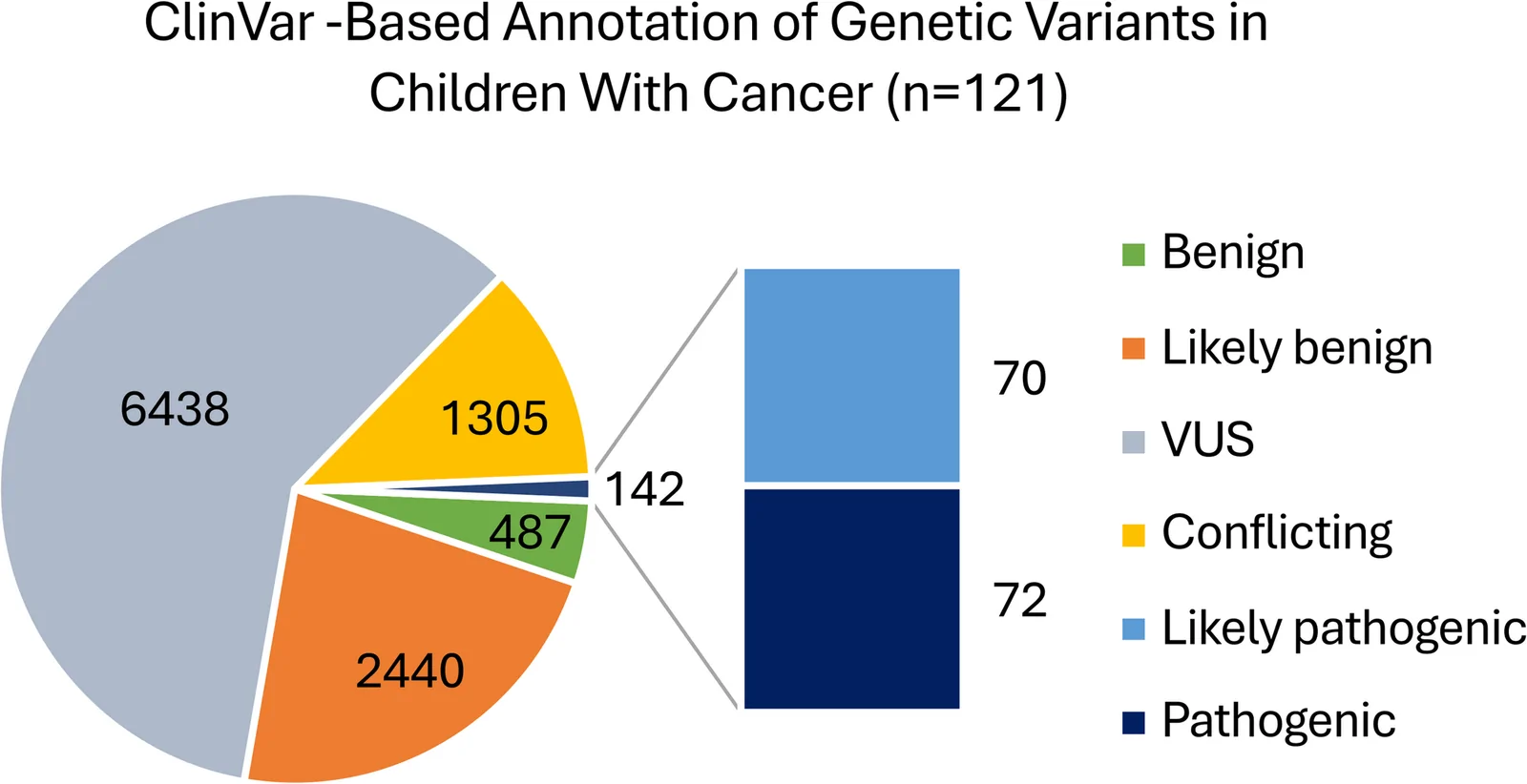
ClinVar annotation of rare variants (MAF ≤ 1%) in *n* = 121 children with cancer. VUS, variants of unknown significance. Conflicting, multiple different annotation labels in ClinVar.

Across a cohort of *n* = 121 children with cancer, 26,495 protein-coding variants with a maximum population allele frequency of less than 0.1% in gnomAD v4.1 were detected. Thereof, 10,812 variants were annotated in ClinVar: 487 were classified as benign, 2,440 as likely benign, 6,438 as variants of uncertain significance (VUS), 1,305 had conflicting interpretations, 70 were classified as likely pathogenic, and 72 as pathogenic (Fig. 5).

Among the 72 pathogenic variants, 9 were annotated as cancer-associated in ClinVar. Of the 70 likely pathogenic variants, 3 were cancer-associated. The cancer-associated variants occurred in genes of the Fanconi anemia pathway (*FANCI*, *FANCD2*, *FANCM*, *FANCA*), in *SEC23B* (two variants, of which one is likely pathogenic), *ERCC6L2* (homozygous), *TSC1*, *WRAP53*, *BLM*, *MRE11* (likely pathogenic), and *ELAC2* (likely pathogenic) (Supplemental **Table S4**).

Of these genes, only *TSC1* is associated with autosomal dominant inheritance and is therefore clinically relevant in the heterozygous state. In addition, the homozygous *ERCC6L2* variant was considered cancer-relevant. The child with the pathogenic autosomal recessive *WRAP53* variant carries an additional *WRAP53* variant on the second allele, currently classified as a VUS, which may likewise have actionable clinical implications. All remaining genes follow autosomal recessive inheritance and were not considered clinically relevant in heterozygous carriers.

These findings are fully concordant with the results reported by *Friedrich et al. (2023)*^52^, as no additional pathogenic or likely pathogenic variants were identified by T-Rex beyond those previously reported. Accordingly, T-Rex achieved 100% sensitivity in detecting all assessable (likely) pathogenic variants from the reference dataset, while generating no false-positive pathogenic calls relative to the prior analysis.

## Discussion

Family-based WES studies, such as Trio designs, are critical for identifying rare germline variants in pediatric diseases, both in individual diagnostic investigations and in research cohorts^8,53^. However, the analysis of Trio WES data often remains dependent on specialized bioinformatics expertise, dedicated computational infrastructure, or access to commercial analysis platforms^14,15^. This can represent a substantial barrier, particularly for smaller clinical institutions without in-house bioinformatics support or access to university-based computational resources^15,23^. Consequently, genomic data analysis and variant prioritization may be delayed while awaiting external bioinformatics support^53^. In research settings, heterogeneous analysis workflows across institutions can further limit the generation of harmonized family-based cohorts^54^.

Although numerous software solutions for WES analysis are available, many consist of multiple independent tools, require substantial programming expertise, or involve complex installation and configuration procedures^14,16,17,20,30,55^. Many packages are operated via command-line scripts and rely on pre-installed software, posing challenges even for experienced bioinformaticians^16,19,56^. Containerization platforms such as Docker simplify software deployment ^19,57^, whereas workflow management systems such as Nextflow and Snakemake enable standardized and reproducible workflow execution^18,58^. While these approaches are highly effective for bioinformatics workflows and deployment on computational clusters, they are primarily designed for users with computational expertise and typically require command-line operation for installation, configuration, and operation^16,18,19,57,58,59^. Galaxy provides a graphical web interface that lowers the barrier to workflow execution; however, users must still identify, select, and configure appropriate workflows for Trio germline WES analysis and possess sufficient domain and bioinformatics expertise to ensure their correct application^60,61^. JWES is a cross-platform application dedicated to WES analysis, however, analyses must be initiated via Java scripts, and the pipeline is restricted to single-sample data rather than Trio studies^62,63^. Commercial solutions for Trio germline WES analysis are also available^21,22^, but their licensing costs, proprietary algorithms, and, in some cases, reliance on external cloud infrastructure may limit accessibility for some academic institutions and clinical settings^23^.

T-Rex was developed to bridge this gap by providing a dedicated Trio WES desktop application that can be operated without programming knowledge. T-Rex is cross-platform and provides a standardized, reproducible, open-source, and user-friendly workflow for Trio WES analysis. Pre-compiled binaries are directly available for download, allowing T-Rex to be launched as a standalone application with minimal setup and without requiring manual installation of dependencies or configuration of a computational environment. T-Rex supports batch execution and job resumption and, for advanced users, can additionally be operated in headless mode with parallel execution on server environments when required.

T-Rex is particularly valuable in settings where bioinformatics support is limited and where sharing raw sequencing data with external analysis facilities is restricted by institutional policies or data protection requirements. By enabling standardized local WES analysis, T-Rex allows clinicians and researchers with diverse technical backgrounds to perform Trio WES analyses independently while facilitating the generation of harmonized multicenter cohorts, an important foundation for advancing artificial intelligence approaches in rare disease research.

A structured comparison of T-Rex with representative workflow-based, graphical, and commercial solutions is provided in Table 3, highlighting the intended application scenarios and strengths of each approach. Among the compared solutions, T-Rex uniquely combines a precompiled, open-source, locally executable end-to-end Trio WES workflow with no programming requirements and integrated inheritance-based filtering and statistical testing.

Additionally, methodologically, T-Rex integrates established best-practice components for germline WES analysis with explicit support for family-based study designs. Unlike pipelines optimized for case-control cohorts, T-Rex incorporates trio-aware variant calling, inheritance-based filtering, and family-based statistical testing. Both case-population and case-parent statistical tests are included, allowing flexible analysis of rare variants across heterogeneous cohorts. The Fisher’s exact test identifies rare variants enriched in affected individuals compared with population controls^41,64^, whereas the TDT identifies variants transmitted from parents to affected offspring more frequently than expected by chance while remaining robust to population stratification^10,65^. For example, a variant enriched in affected individuals by Fisher’s exact test but not supported by TDT may reflect either a population-associated signal or a true risk variant whose transmission pattern is obscured by incomplete penetrance, modest effect size, or limited statistical power^66^. Thus, both tests together provide complementary insights into genetic association signals.

**Table 3:** Structured comparison of T-Rex with representative workflow-based, graphical, and commercial solutions, focusing on intended application scenarios, required bioinformatics and programming expertise, and installation complexity.

Feature	T-Rex	Galaxy^60^	Snakemake^58^/ Nextflow^18,59^	Clinical Interpretation Platforms	Commercial Analysis Platforms
Representative examples	T-Rex	Exome Seq Training^67^	Sarek^68^, wes_gatk^69^	ELLA^70^, UniVar^71^, ACVI-Med^72^	VarSeq Suite^21^, Franklin^22^
Main purpose	Desktop application for local, standardized Trio WES analysis in settings without dedicated bioinformatics infrastructure	Interactive, web-based workflow execution and analysis environment	Large-scale and reproducible workflow automation	Variant interpretation after variant calling	Routine clinical genomics with institutional infrastructure
Typical users	Clinicians/researchers without programming expertise	Users with intermediate bioinformatics knowledge	Bioinformaticians	Clinicians/geneticists	Clinical laboratories
User interface	GUI	Web-based workflow editor	Command line	Web-based GUI/ command line	GUI/ web-based
Required programming knowledge	None	Low-Medium	High	Low-Medium	None
Required bioinformatics knowledge	Low	Medium	High	Medium	Low-Medium
Installation complexity	Low (pre-compiled application)	Medium	Medium-High	Platform-dependent	*Usually handled by provider*
Local execution (privacy)	Yes	Possible (local deployment)	Yes	Platform-dependent	Platform-dependent
Cluster/ HPC compatibility for human germline data	Possible via headless execution; no direct GUI-HPC integration	Yes, if deployed on approved infrastructure	Yes, if executed on approved infrastructure	Usually limited	Provider-dependent
End-to-end FASTQ-to-results germline WES workflow	Yes	Workflow-dependent	Workflow-dependent	No	Yes/ platform-dependent
Trio-aware analysis	Yes	Workflow-dependent	Workflow-dependent	Interpretation-focused	Yes/ platform-dependent
Inheritance-based Filtering	Yes	Workflow-dependent	Workflow-dependent	Yes/ platform-dependent	Yes/ platform-dependent
Rare Variant Filtering/ Statistical Testing	Yes	Limited/ Workflow-dependent	Limited/ Workflow-dependent	No	Variable
Supported variant classes	SNPs, Indels	Tool-dependent	Workflow-dependent	Tool-dependent	Usually broad
Open-Source/ licensing	Open source, no licensing costs	Open source	Open source	Variable	Commercial licensing

Variant detection is performed using an ensemble strategy combining GATK HaplotypeCaller v4 and VarScan2, leveraging complementary strengths while intersecting calls to prioritize specificity and reproducibility, which is particularly important in rare disease studies. The performance of this dual-caller approach was benchmarked on the Ashkenazim Trio reference dataset from GIAB. Compared to the GIAB high-confidence truth set for HG002 (child), the dual-caller consensus workflow substantially reduced false positives (175 FPs) relative to the individual callers, while maintaining acceptable sensitivity (91.1%). In contrast, GATK HaplotypeCaller v4 alone achieved higher sensitivity (95.5%) but generated 916 false positives, whereas VarScan2 exhibited slightly lower precision (99.0%; 204 FPs) and similar sensitivity (91.1%). These results demonstrate that the dual-caller strategy effectively prioritizes high-confidence variant calls and reduces spurious calls, supporting its use in research and clinical contexts where precision in rare variant detection is critical. For studies where maximal sensitivity is required, the workflow can be configured to use a single caller (GATK HaplotypeCaller v4) rather than the dual-caller consensus.

Beyond its technical performance, T-Rex directly addresses the strategic objectives of national and international digital health initiatives, such as the German CORD-MI (Collaboration on Rare Diseases) project within the Medical Informatics Initiative (MII) and the European Solve-RD consortium^73,74^. These programs aim to improve patient care by overcoming the fragmentation of genomic data across clinical centers. While these initiatives provide the legal and ethical frameworks for data sharing, T-Rex provides the necessary technical substrate for decentralized data harmonization. By enabling sites to execute identical, trio-aware pipelines locally, T-Rex facilitates a ‘federated analysis’ model consistent with GA4GH standards — where the analysis is brought to the data rather than the data to a central repository^75^. This approach ensures that even institutions with limited bioinformatics infrastructure can contribute high-quality, pre-processed variant calls to multicenter cohorts, fulfilling the mandate of the German Federal Ministry of Education and Research for privacy-compliant, cross-institutional research to improve diagnostic yields in rare disease populations.

In the here tested pediatric cancer re-analysis cohort, the tumor distribution is closely aligned with the expected distribution based on the European Cancer Registry, except for nephroblastoma (*n* = 3), which was slightly underrepresented in the study (n_expected_=6)^76^. Our analysis yielded ~ 26,495 rare protein-coding variants, including 12 ClinVar-classified (likely) pathogenic cancer-associated variants. T-Rex successfully detected all previously reported (likely) pathogenic variants and did not identify additional pathogenic calls relative to the reference analysis, indicating 100% sensitivity and high specificity with respect to the published dataset, without compromising the detection of clinically relevant variants. User testing across different centers confirmed that T-Rex can be rapidly adopted by users with diverse technical backgrounds, and performance evaluation demonstrated linear time complexity and constant memory usage, enabling execution on standard local hardware.

T-Rex Limitations: T-Rex currently supports short-read Illumina WES and germline variant detection. Thus, whole-genome sequencing, long-read technologies, and somatic variant calling are not yet supported. The intersecting dual-variant-caller strategy prioritizes high-confidence calls but may reduce sensitivity, particularly for low-level mosaic variants or variants present only in one caller. For studies where maximal sensitivity is required, the workflow can be configured to use a single caller (GATK HaplotypeCaller) instead of the dual-caller consensus. Additionally, pathogenicity assessment relies on external annotation resources, which are subject to ongoing revision. Hence, final variant assessment must still be performed by a trained geneticist. Although T-Rex supports headless execution and parallel analysis on server environments, it currently does not provide direct integration of the graphical user interface with HPC cluster schedulers or remote server infrastructures. Advanced users can deploy T-Rex on servers and configure such environments manually, but this requires additional technical expertise.

In conclusion, T-Rex is among the first locally executable, cross-platform WES analysis platforms specifically designed for Trio data that can be operated and installed without programming expertise. It integrates standardized, reproducible workflows with trio-aware variant calling and family-based statistical testing, accurately recovering known pathogenic variants without introducing additional false positives. The GIAB validation confirms the reliability of the dual-caller strategy for high-confidence variant detection, supporting its use in clinical and research applications. By removing technical barriers and enabling decentralized analysis, T-Rex facilitates collaborative studies across clinical settings and enables the discovery of rare pathogenic variants and the genetic mechanisms underlying disease in Trio WES data.

## Supplementary Information

Below is the link to the electronic supplementary material.


Supplementary Material 1


## Data Availability

Variant pathogenicity annotations were obtained from the ClinVar VCF dataset (GRCh38, release 20.07.2026), available from the National Center for Biotechnology Information (NCBI). Allele frequency data were derived from gnomAD v4.1 exomes (GRCh38 assembly).Whole-exome sequencing reference datasets for the Ashkenazim Trio (HG002, HG003, HG004), generated by the Genome in a Bottle (GIAB) Consortium, were downloaded from the European Nucleotide Archive (accessions SRR2962669, SRR2962692, SRR2962694).The raw data sets generated and/or analyzed during this study are not publicly available because of personal data restrictions but are available from the corresponding authors on request. This excludes any individual personal/ clinical data of the individuals, which would endanger their anonymity.T-Rex is available as a pre-compiled application via Zenodo (https://zenodo.org/records/19135261), along with accompanying test datasets. The source code is publicly available at GitHub (https://github.com/SaraLuisaReh/trex). T-Rex is compatible with macOS (ARM64), Linux (x86; Ubuntu ≥24), and Windows (≥11 via WSL2).

## Code availability

The source code is available on GitHub (https://github.com/SaraLuisaReh/trex). The fully precompiled app is available on Zenodo (https://doi.org/10.5281/zenodo.19135261).
