# Supplementary material for: T-rex: standardized analysis of germline variants in whole-exome sequencing trios
Source: Sci Rep. 2026 Aug 31;16:27284. doi: 10.1038/s41598-026-67762-w (PMC13529699; doi:10.1038/s41598-026-67762-w)
Supplement: Supplementary file 1 [file 41598_2026_67762_MOESM1_ESM.docx]

**Supplementary Figures and Materials**

**Supplementary Section 1**

**User Instructions for User Acceptance Testing**

Please familiarize yourself with the platform. Read through the settings and the tasks below.

Setting:

You work in a clinic/research department that has sequenced the DNA of exomes from five children with cancer and their healthy parents. The raw sequencing data (FASTQ files) are stored on a hard disk. For a cooperation project with other clinics, you are asked to carry out the following tasks.

Tasks:

a)	Use the FASTQ files and filter for all variants with a maximum allele frequency of 80% in the European reference population.

b)	Use the CSV file produced in Task a) to filter for variants with a maximal allele frequency of 1%.

c)	Use the BAM file to filter for only protein-coding variants with a maximal allele frequency of 5% in the Non-Finnish European population.

d)	You have received Trio variant calling files (VCF Files) from a cooperating institute, and you are asked to filter for homozygous variants with a maximal allele frequency of 5% in the European population, which are significant in the Transmission Disequilibrium Test.

**Supplementary Table S3**

| **Test person** | **Profession** | **Age** | **Task 1** | **Task 2** | **Task 3** | **Task 4** |
| --- | --- | --- | --- | --- | --- | --- |
| 1 | Research Scientist | 24 |  |  |  |  |
| 2 | Medical Doctor | 29 |  |  |  |  |
| 3 | Medical Doctor | 53 |  |  |  |  |
| Intervention: Replace the tab view, where the user can choose between multiple files and a single file, with a single input field, which automatically recognizes the type of entry. | | | | | | |
| 4 | Medical Doctor | 29 |  |  |  |  |
| 5 | Research Scientist | 31 |  |  |  |  |
| 6 | Medical Doctoral Candidate | 24 |  |  |  |  |
| 7 | Research Scientist | 24 |  |  |  |  |
| 8 | Medical Doctor | 58 |  |  |  |  |
| Intervention: Open the subsection, which lists the additional filtering options, from the beginning. | | | | | | |
| 9 | Research Scientists | 62 |  |  |  |  |
| 10 | Medical Doctoral Candidate | 22 |  |  |  |  |
| Intervention: Add a label on the top of the page that reminds the user to select the correct file format. | | | | | | |
| 11 | Research Scientist | 25 |  |  |  |  |
| 12 | Research Scientist | 24 |  |  |  |  |
| 13 | Medical Doctor | 27 |  |  |  |  |

Table S3: User testing results and platform adjustments during user acceptance testing.

**Supplementary Table S4**

| Cancer Type | Gene | Variant | Effect |
| --- | --- | --- | --- |
| MDS | ERCC6L2^a^ | 9:95955996_C/T | stop_gained |
|  | FANCI | 15:89315318_C/T | stop_gained |
|  | FANCD2 | 3:10092206_G/A | stop_gained |
|  | WRAP53^c^ | 17:7701738_G/T | missense_variant |
| Leukemia | FANCM | 14:45166959_C/T | stop_gained |
|  | ELAC2^b^ | 17:13017778_G/A | missense_variant |
| Lymphoma | MRE11^b^ | 11:94447276_G/A | stop_gained |
| Sarcoma | FANCA | 16:89799197_C/A | stop_gained |
| Germ Cell Tumor | SEC23B | 20:18515695_G/A | missense_variant |
|  | SEC23B^b^ | 20:18510875_C/T | missense_variant |
| Brain Tumor | TSC1^c^ | 9:132905615_G/A | stop_gained |
|  | BLM | 15:90761015_C/T | stop_gained |

Table S4: Rare pathogenic variants identified in the Trio cohort (n = 121, MAF ≤ 0.1%) that are associated with cancer according to ClinVar. Allele frequencies were evaluated using the European reference population of gnomAD v4.0 (n = 582,716).

a Homozygous variant in the affected child.

b Variant annotated as likely pathogenic.

c Autosomal dominant.

In total, 12 (likely) pathogenic cancer-associated variants were identified. Thereof, three were cancer-relevant (denoted with a and c).

MDS, myelodysplastic syndrome.
